# Lung erosion following adjuvant immunotherapy with pembrolizumab: a case report

**DOI:** 10.1186/s13256-023-04162-y

**Published:** 2023-10-07

**Authors:** Lowell Leow, Manisha Anbudurai, Li Yue, John Kit Chung Tam

**Affiliations:** 1 Department of Cardiac, Thoracic and Vascular Surgery, National University Heart Centre Singapore, Singapore, Singapore; 2 Department of Surgery, Yong Loo Lin School of Medicine, National University Singapore, 5 Lower Kent Ridge Road, Singapore, 119074 Singapore

**Keywords:** Pembrolizumab, Adjuvant therapy, Lung surgery, Lung cancer, Case report

## Abstract

**Background:**

Pembrolizumab as immunotherapy is increasingly used in adjuvant, neoadjuvant, and standalone therapy and has been described as safe. We share an experience of lung erosion post-thoracic surgery with the use of adjuvant pembrolizumab.

**Case presentation:**

A 65-year-old Chinese gentleman with metastatic renal cell carcinoma underwent lung metastasis resection and presented with delayed onset pneumothorax while on adjuvant pembrolizumab. Failure of conservative management warranted repeat surgical intervention, and intraoperative findings showed erosion of staple lines possibly caused by poor healing associated with pembrolizumab.

**Conclusion:**

Adjuvant pembrolizumab may impair wound healing, including stapler line healing. Presentation of delayed pneumothorax in a post-surgical patient undergoing immunotherapy should warrant early surgical intervention.

## Introduction

Pembrolizumab is a programmed death-ligand 1 (PD-L1) inhibitor that is increasingly used for lung cancer. While it is mainly used in non-resectable disease, it has been shown to be safe and well tolerated as neoadjuvant therapy for lung cancer, with no incidences of 90-day mortality but a higher rate of conversion for fibrosis [1, 2]. While there have been reports of bronchopleural fistula and pneumonia following surgery after neoadjuvant pembrolizumab [3, 4], we were unable to find similar complications of impaired stapler line healing and lung erosion [5] to what our patient has experienced. Other checkpoint inhibitors such as nivolumab have been associated with lung toxicity [6].

## Case

**Fig. 1:**
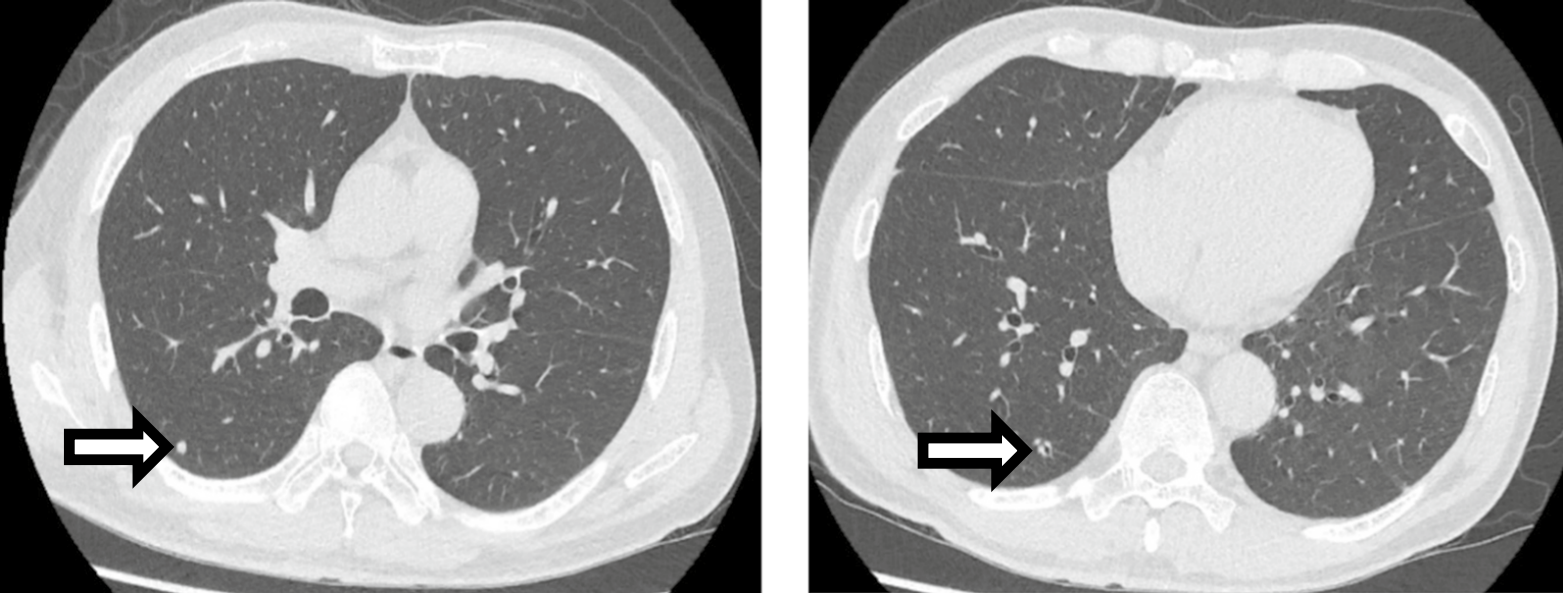
Right lower lobe superior segment lesion (left) and right lower lobe basal segment lesion (right). Arrows indicate lesions

The patient was a 65-year-old Chinese gentleman with a background of anxiety with palpitations and stage 3A (pT3aN0M0) grade 4 left clear cell renal cell carcinoma for which he had undergone a left nephrectomy 1 year prior. He did not have a history of diabetes, metabolic disease, smoking, or malnutrition. He had not received any anti-vascular endothelial growth factor monoclonal antibody post-surgery. Staging scans prior to his urological surgery did not demonstrate any lung lesions. However, subsequent surveillance scans revealed right lower lobe lesions that progressed over a period of 6 months from 0.3 to 0.5 cm (Fig. 1). He was thus referred for resection of the right lung nodules for diagnostic and therapeutic intent and had agreed to proceed with surgery.

He underwent a right uniportal video-assisted thoracoscopic surgery with radiologically guided resection of the lung lesions. Prior to surgery, the lesions were localized with computed-tomography-assisted placement of metallic coils by interventional radiologists. This facilitated localization of the lesions using intraoperative imaging. The patient had an uneventful postoperative recovery. Histology confirmed that one of the lesions was metastatic clear cell carcinoma of renal origin. The patient was referred to oncology and was recommended for 1 year of adjuvant pembrolizumab treatment [7].

**Fig. 2:**
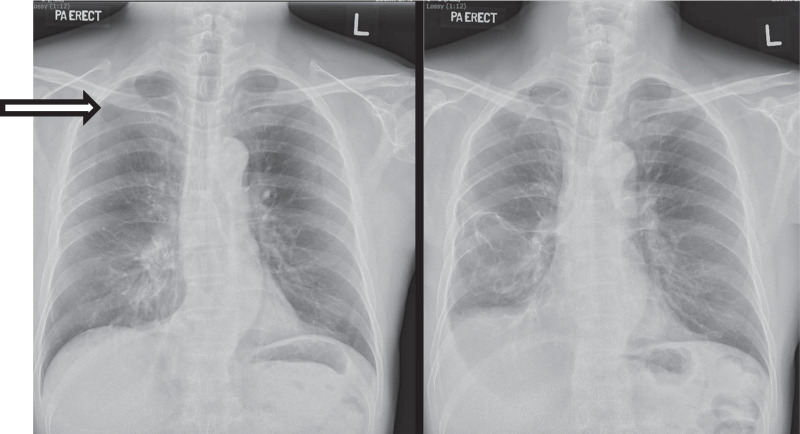
Apical pneumothorax (left) resolved (right) after superior segmentectomy surgery. Arrow indicates pneumothorax

He was initiated on pembrolizumab and tolerated three cycles of treatment with a dose of 200 mg each. However, 4 months after surgery, he complained of worsening effort tolerance and was noted to have a right apical pneumothorax that was increasing in size (Fig. 2). He was admitted for chest drain insertion. Despite conservative measures, there was persistent air leak, and the pneumothorax did not resolve even after a month on Heimlich valve. He eventually agreed to surgical intervention for his persistent pneumothorax.

**Fig. 3:**
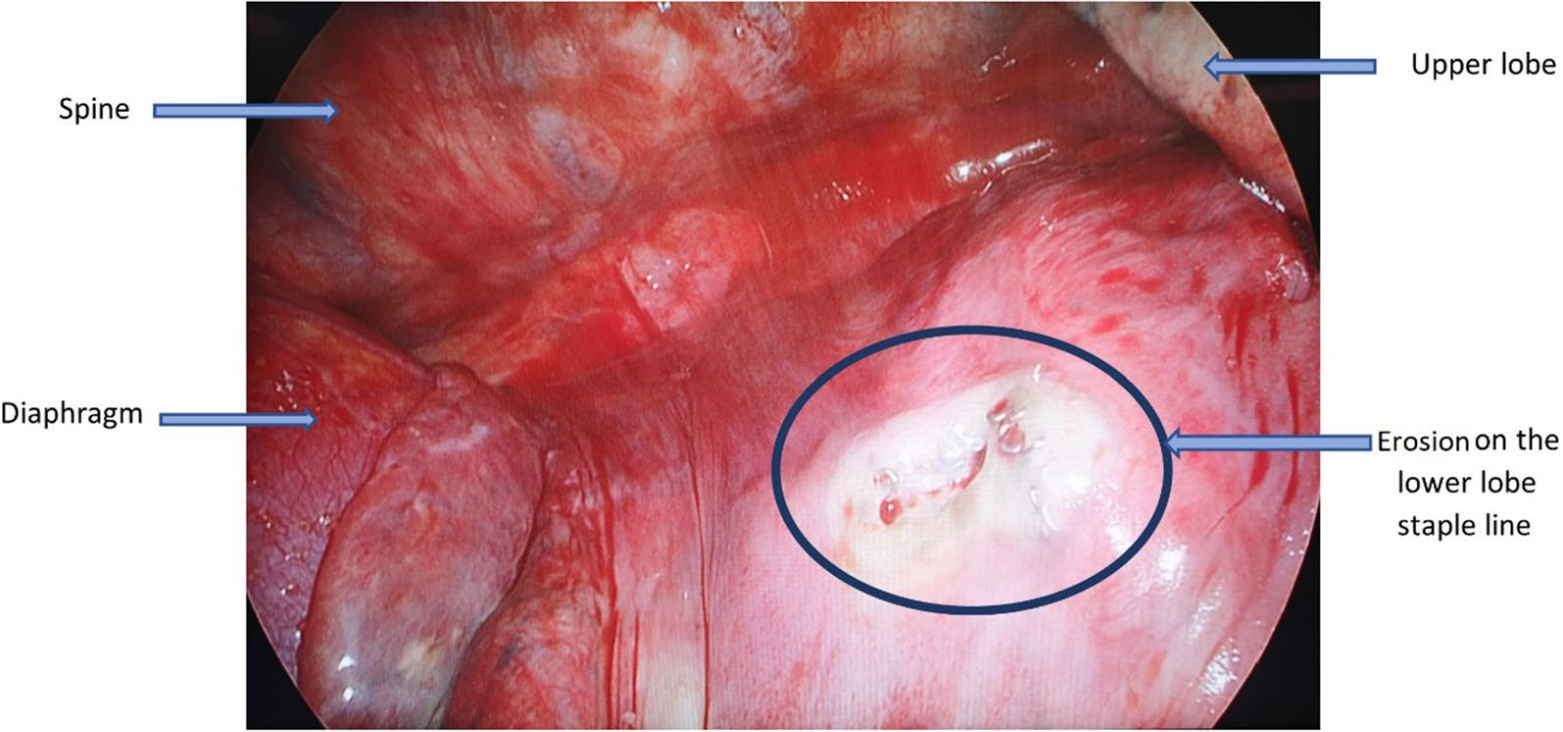
Intraoperative photograph of erosion on lower lobe stapler line

He underwent a right lateral mini-thoracotomy adhesiolysis and superior segmentectomy. Expectedly, there were dense adhesions from the previous surgery; hence, a mini-thoracotomy was performed. An area of lung erosion was seen on the lower lobe stapler line, which had active bubbling upon underwater provocation. The rest of the stapler line had been epithelized with pleura. The superior segment was resected anatomically to eliminate this erosion (Fig. 3). The patient recovered uneventfully and has since been well. Histology did not show any recurrence of malignancy along the stapler line, but there was a pleural defect with fibrosis measuring 2.3 × 1.0 × 0.8 cm. He has since been restarted on pembrolizumab, after receiving counseling on the benefit of disease control versus the risk of recurrent pneumothorax.

## Discussion

There have been retrospective studies done detailing the side effect profile of pembrolizumab. The most common immune-related adverse events are pneumonitis and dermatological rashes [8, 9]. These usually occur early on after initiation of immunotherapy [8]. In our patient, the pneumothorax occurred within 3 months of initiating pembrolizumab. There also appears to be an increased risk of adverse events associated with increasing number of doses [9]. Our patient had three doses of pembrolizumab before the detection of pneumothorax, but due to a lack of symptoms, we cannot determine precisely when the adverse event occurred. Immunotherapy has been shown to cause wound complications in other cancers [10], although pembrolizumab seems to be the safest in profile [11]. Hence, given the efficacy of pembrolizumab and its relatively low rate of adverse events, it remains a popular choice as neoadjuvant and adjuvant systemic therapy, especially for malignancies with high PD-L1 expression [1, 2].

**Fig. 4:**
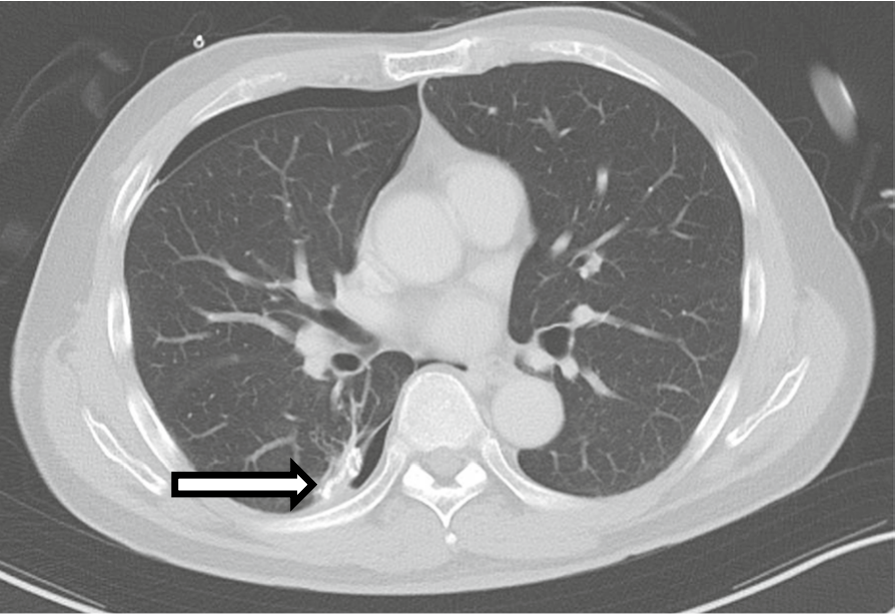
Abnormal healing around staple line in superior segment right lower lobe, with concomitant right pneumothorax. Arrow indicates stapler line

Pembrolizumab has been described to be associated with pneumothorax, possibly related to a high expression of PD-L1 in lung cancers [12]. Other immune checkpoint inhibitors have also been found to cause pneumothorax most significantly at regions not involved with malignancy, which suggests that the pneumothorax is related directly to drug- or immune-related pneumonitis [4, 13]. Histology of our patient excluded recurrence malignancy along the stapler line as a cause for the pneumothorax. Instead, the effect of pembrolizumab was likely impairment of stapler line healing. In a similar case, a woman had repeated episodes of secondary pneumothorax on nivolumab for metastatic renal cell carcinoma [13]. It is worth exploring whether the use of immune checkpoint inhibitors specifically for renal cell carcinoma predisposes patients to this respiratory adverse event. In a report on the use of pembrolizumab in the neoadjuvant setting, early bronchopleural fistula occurred but resolved with prolonged conservative management [3]. It would be reasonable to consider the potential effect of pembrolizumab on the healing process that may result in the bronchopleural fistula. We hypothesize that, in our patient, given the paucity of other potential confounding medical issues, immunotherapy may have affected the healing of the stapler line and eventually led to an erosion that resulted in the pneumothorax (Fig. 4). Perhaps a longer interval between surgery and initiation of pembrolizumab could have avoided this complication.

## Conclusion

We share our unique experience of a delayed postoperative complication with the adjuvant use of pembrolizumab, which raises concerns on healing impairment post-operatively. Delayed presentation of pneumothorax in a patient post-lung-resection on immunotherapy should prompt clinicians to consider early surgical intervention for definitive management.

## Data Availability

Data sharing is not applicable to this article, as no datasets were generated or analyzed during the current study.

## Abbreviation

PD-L1: Programmed death-ligand 1
